# Discovery of FNDR-20123, a histone deacetylase inhibitor for the treatment of *Plasmodium falciparum* malaria

**DOI:** 10.1186/s12936-020-03421-3

**Published:** 2020-10-12

**Authors:** Vijay Potluri, Radha K. Shandil, R. Gavara, Ganesh Sambasivam, Brice Campo, Sergio Wittlin, Shridhar Narayanan

**Affiliations:** 1 Foundation for Neglected Disease Research, Bengaluru, India; 2 Anthem Biosciences Private Limited, Bengaluru, India; 3 Medicines for Malaria Venture, Geneva, Switzerland; 4 Swiss Tropical and Public Health Institute, Basel, Switzerland

**Keywords:** Malaria, *Plasmodium falciparum*, Histone deacetyl

## Abstract

**Background:**

Emergence of anti-malarial drug resistance and perpetual increase in malaria incidence necessitates the development of novel anti-malarials. Histone deacetylases (HDAC) has been shown to be a promising target for malaria, despite this, there are no HDAC inhibitors in clinical trials for malaria treatment. This can be attributed to the poor pharmacokinetics, bioavailability and selectivity of the HDAC inhibitors.

**Methods:**

A collection of HDAC inhibitors were screened for anti-malarial activity, and the best candidate was profiled in parasite-killing kinetics, growth inhibition of sensitive and multi-drug resistant (MDR) strains and against gametocytes. Absorption, distribution, metabolism and excretion pharmacokinetics (ADME-PK) parameters of FNDR-20123 were determined, and in vivo efficacy was studied in a mouse model for *Plasmodium falciparum* infection.

**Results:**

A compound library of HDAC inhibitors (180 in number) was screened for anti-malarial activity, of which FNDR-20123 was the most potent candidate. The compound had been shown to inhibit *Plasmodium* HDAC with IC_50_ of 31 nM and human HDAC with IC_50_ of 3 nM. The IC_50_ obtained for *P. falciparum* in asexual blood-stage assay was 42 nM. When compared to atovaquone and pyrimethamine, the killing profiles of FNDR-20123 were better than atovaquone and comparable to pyrimethamine. The IC_50_ values for the growth inhibition of sensitive and MDR strains were similar, indicating that there is no cross-resistance and a low risk of resistance development. The selected compound was also active against gametocytes, indicating a potential for transmission control: IC_50_ values being 190 nM for male and > 5 µM for female gametocytes. FNDR-20123 is a stable candidate in human/mouse/rat liver microsomes (> 75% remaining post 2-h incubation), exhibits low plasma protein binding (57% in humans) with no human Ether-à-go–go-Related Gene (hERG) liability (> 100 µM), and does not inhibit any of the cytochrome P450 (CYP) isoforms tested (IC_50_ > 25 µM). It also shows negligible cytotoxicity to HepG-2 and THP-1 cell lines. The oral pharmacokinetics in rats at 100 mg/kg body weight shows good exposures (C_max_ = 1.1 µM) and half-life (T_1/2_ = 5.5 h). Furthermore, a 14-day toxicokinetic study at 100 mg/kg daily dose did not show any abnormality in body weight or gross organ pathology. FNDR-20123 is also able to reduce parasitaemia significantly in a mouse model for *P. falciparum* infection when dosed orally and subcutaneously.

**Conclusion:**

FNDR-20123 may be a suitable candidate for the treatment of malaria, which can be further developed.

## Background

Malaria remains one of the most severe and life-threatening infectious diseases, imposing a substantial socio-economic burden in low- and middle-income countries. Every year it accounts for almost 500,000 deaths, with over 200 million cases of occurrence worldwide [1]. India, along with Africa, carries almost 80% of the global malaria burden. The mosquito-borne disease is transmitted to humans through *Plasmodium*-infected female *Anopheles* mosquito. Amongst the various *Plasmodium spp*., *Plasmodium falciparum* is known to be the most virulent and life-threatening form, prevailing in Southeast Asia and Africa. Most parts of India have also shown high transmission of *Plasmodium vivax,* but recent reports suggest the emergence of chloroquine-resistant *P. falciparum* in several regions [2]. At present, artemisinin derivatives or quinine-based compounds are used for treating malaria-infected patients. Acute treatment of non-falciparum malaria still requires the use of chloroquine, which is seldom used as a preventive treatment due to resistance developed against it. There is an emergent need for developing prompt treatment methods against multidrug-resistant strains of *P. falciparum.*

Epigenetic mechanisms have been promising therapeutic targets for a variety of diseases ranging from cancer, cardiovascular diseases, inflammation and infection. The interaction between the histone acetyltransferases (HATs) and the histone deacetylases (HDACs) facilitates the structure of the intact DNA. The epigenetic mechanisms play a role in chromatin modifications, gene mutations in DNA, inactivation of DNA repair mechanisms, activation of oncogenes, and apoptosis. Following this, a new class of chemical compounds known as HDAC inhibitors were identified with a potential to target *Plasmodium* and other Apicomplexan parasites more than a decade ago. Several drugs of the same class, such as vorinostat, panobinostat, belinostat, and romidepsin, have already been approved for the treatment of various types of cancers [11].

HDACs are known to remove acetyl groups from histones and other proteins [3]. Regulation of numerous essential biological processes in eukaryotes, including transcriptional regulation [4], cell cycle progression [5] and apoptosis [6] is a contribution of these post-translational modifications. Certain human diseases, such as cancer, are caused as a result of aberrant expression of these proteins, thus making these epigenetic regulatory enzymes as ‘druggable’ targets [3, 7, 8]. The HDAC enzymes which are known as lysine deacetylases (KDACs) in parasites have been identified as an important target for treating drug-resistant parasitic infections [12].

The HDAC family consists of 18 different isoforms: HDAC (1–11) and sirtuin (SIRT) (1–7). They are classified into Zinc-dependent (Class 1, Class IIa, IIb and IV) and NAD-dependent (Class III.

A systematic study conducted by Wang et al. showed that subtle differences in the chemical structure can alter the functional activity of the molecule depending upon the different isoforms that a molecule acts on or the cellular distribution of the target enzyme [32].

The *Plasmodium falciparum* HDAC (*Pf*HDAC) family consists of at least five isoforms with the *P. falciparum* HDAC1 being identified as the primary target of most anti-malarial molecules [13, 14]. Following this, while studies exhibit that *Pf*HDAC inhibition inhibits asexual *P. falciparum* in erythrocytes [9], there is also evidence of HDAC inhibitors showing activity against multidrug-resistant clinical isolates of *P. falciparum* and *P. vivax* [15].

Reports also suggest that the treatment of *P. falciparum* parasites with HDAC inhibitors results in genome-wide transcriptional alterations [16–18] and altered *Pf*HDAC1 expression has been found in *P. falciparum* parasite lines with reduced clinical susceptibility to artemisinin [19].

The structural diversity of HDAC inhibitors is limited to a few classes, such as cyclic peptides (apicidin and its analogues), hydroxamates such as suberoylanilide hydroxamic acid (SAHA, vorinostat), trichostatin A (TSA), WR301801 and benzamides (MS-275). Apicidin, a cyclic tetrapeptide was found to have an IC_50_ of 200 nM in *P. falciparum* but was not selective. However, replacing the indole in apicidin with quinolone increased the selectivity (up to ~ 200-fold) for *P. falciparum* in whole-cell assay compared to activity obtained for mammalian cells. Hydroxamate-based HDAC inhibitors showed more promising in vitro profiles. This class of inhibitors includes the class I/II HDAC inhibitors TSA, SAHA, vorinostat and a sulfonylpyrrole hydroxamate (4SC-201, resminostat), with vorinostat being the HDAC inhibitors approved for cancer therapy. Many of the compounds in clinical development have limitations, e.g., poor selectivity for human HDACs and/or low bioavailability, hence preventing these candidates from being considered for anti-malarial therapy.

Recently, several hydroxamic acid-based compounds with higher in vitro inhibitory potency against *P. falciparum* parasites than vorinostat, and with varying improvements on selectivity, were reported [13, 20]. The compounds were identified by screening with variations to the basic structure of HDAC inhibitors. They comprise a small zinc-binding group (ZBG) that accesses the active site zinc ion, a linker region capable of fitting the narrow, hydrophobic, tubular cavity leading from the ZBG to the HDAC surface, and a capping group that blocks the entrance to the active site cavity. Hydroxamate compounds based on an l-cysteine (thioether in the linker region) or 2-aminosuberic acid (methylene group in the linker region) scaffold show similar in vitro anti-parasitic potency (IC_50_ < 200 nM), however better selectivity for *P. falciparum versus* mammalian cells was generally observed for the 2-aminosuberic acid (ASA) compounds. Compounds (three in number) from phenyl-thiazolyl-hydroxamate-based HDAC inhibitor class were highly potent (IC50 < 3 nM) with high selectivity indices of > 600. WR301801, a lead compound from this panel, had an IC_50_ of 0.5–1.5 nM against several drug-resistant lines of *P. falciparum,* hyperacetylated *P. falciparum* histones in situ, and inhibited deacetylase activity in *P. falciparum* nuclear extracts [21]. Thus, there is substantial evidence that suggested the use of HDAC inhibitors against multidrug-resistant clinical isolates of *P. falciparum* and *P. vivax* in anti-malarial therapy [15]. The use of HDAC inhibitors in anti-malarial therapy is also supported by overwhelming evidence wherein; *Pf*HDAC1 inhibitors have shown activity against three life-cycle stages of the parasite (asexual, exo-erythrocytic, gametocyte stages) [9, 10]. However, the existing HDAC inhibitors at present are administered intravenously owing to poor pharmacokinetic properties. Thus, the use of the inhibitors in anti-malarial therapy is limited to their pharmacokinetics, bioavailability and selectivity. Also, at present, as far as the author’s knowledge goes, there are no other HDAC inhibitors in development for the treatment of malaria. The present study reports the identification and development of FNDR-20123, a potent *Pf*HDAC inhibitor for anti-malarial therapy. Pre-clinical studies carried out also indicate that FNDR-20123 inhibits Class III HDAC isoforms at nanomolar concentrations.

## Methods

### Culture start-up and maintenance

*Plasmodium falciparum* 3D7 strain was used for the *P. falciparum* asexual blood-stage (ABS) assay, for which the cells were resuscitated from the stabilate and maintained at 5% haematocrit. Red blood cells obtained from the hospital were used to maintain the culture at 1% parasitaemia while screening.

### HDAC activity screening

HDAC inhibition screening was performed using a fluorescence-based assay with a fluorescent substrate (Boc-Lys (Ac)-AMC Substrate). HeLa nuclear extract was used as the enzyme source. For selected compounds, IC_50_ (50% HDAC inhibitory concentration) was determined by testing in a broad concentration range of 0.001, 0.01, 0.1, 1 and 10 µM [22].

The assay was performed in 96-well black microplates, and the total volume of the assay was set at 100 µl. Briefly, HeLa nuclear extract was diluted with HDAC assay buffer (final concentration of 30 µM), containing 25 mM Tris/Cl, pH 8.0, 137 mM NaCl, 2.7 mM KCl, and 1 mM MgCl_2_. The enzyme mixture was prepared by adding 10 mM of the diluted enzyme (HeLa nuclear extract) to 30 µM of HDAC buffer. From the enzyme mixture, 40 µl was taken and mixed with 10 µl of test compound (final concentration from 0.01 to 10 µM) or vehicle (control). The final mixture (50 µl) was added to each well which was then pre-incubated at 37 °C for 10 min. The HDAC reaction was started by adding 50 µl of HDAC substrate: Boc-Lys (Ac)-AMC (Anaspec, Inc Fremont, Calif., USA). The plate was incubated at 37 °C for 45 min. Trypsin stop solution (50 µl) was added to the well, and the plate was further incubated at 37 °C for 15 min to stop the reaction. The release of AMC was monitored by measuring the fluorescence at an excitation wavelength of 360 nm and an emission wavelength of 460 nm. Buffer and substrate alone served as blank.

Isoform selectivity was tested using recombinant HDAC isoforms (Biomol, USA). FNDR-20123 was tested against human HDAC1, HDAC2, HDAC3, HDAC6, and HDAC8 isoforms.

The activity of FNDR-20123 against *Pf*HDAC was assessed with a HDAC fluorescent activity assay kit (BPS-Bioscience’s pf-HDAC1, Malaria,His-tag,FLAG-tag FNDR-20123 was dissolved in 100% DMSO and stored in − 20 °C until use. Enzyme concentration (*Pf*HDAC1) was optimized to 4 ng/µL to get detectable activity. TSA was used as a control in the assay to determine the inhibitory activity. Briefly, HDAC assay buffer (25 mM Tris/Cl, pH 8.0, 137 mM NaCl, 2.7 mM KCl, and 1 mM MgCl_2_), BSA, HDAC substrate and *Pf*HDAC1 enzyme were mixed in an amber colour 96-well plate and incubated at 37 °C for 30 min.

After incubation, the reaction was stopped by adding 50 µl of HDAC assay developer and the plate was incubated further at room temperature for 30 min. The fluorescence developed was measured with excitation at 350–380 nm and emission at 440–460 nm.

### *Plasmodium falciparum* asexual blood-stage (ABS) assay

*Plasmodium falciparum* 3D7 cells were used as a target strain for the assay. Mefloquine (Sigma-Aldrich) was used as a standard inhibitor. At day 1, 250 ml of 10 mM mefloquine was added to columns 12 and 24 of the sterile 384-well black, clear-bottom, cell culture assay plates followed by compound curves which were added using Echo. The *P. falciparum* 3D7 cells were counted and 20 ml of culture was prepared at 5% haematocrit, 0.3% parasitaemia (as one batch, i.e., 12 plates, prepare 260 ml, to allow for WellMate prime volume) for each plate. From this, 50 µl of culture was added to all wells on all plates using WellMate with small bore tubing on full (S-1) speed. The plates were placed on the bottom shelf of the incubator, with a maximum stack height of 4 plates, preferring the front of the shelf and were incubated for 72 h at 37 °C in an atmosphere of special gas mix (1% O_2_, 3% CO_2_, balance N_2_).

At day 4, SybrGreen/lysis buffer was prepared by diluting defrosted SybrGreen aliquots as required (20 μl of 10,000 × req. for 12 plates) to 3× with lysis buffer (70 ml for 12 plates). Ten μl of the prepared buffer was added to each well on each of the assay plates and incubated overnight in the dark at room temperature. The plates were read on Victor plate reader using ‘384sybrgreen’ protocol (excitation 485 nm, emission 528 nm) after which the plate contents were aspirated into 5% Virkon, and disposed of after autoclaving. Percentage inhibition for each test compound was calculated using the following equation:

$$\% {\text{ Inhibition}}\, = \, 100\, - \,\left( {{\text{TEST COMPOUND}}\, - \,{\text{BLANK}}} \right)/\left( {{\text{NO INHIBITION}}\, - \,{\text{BLANK}}} \right)\,*\, 100)$$

EC_50_ values for standard inhibitors were determined using non-linear regression curve-fitting within Activity Base data analysis template.

FNDR-20123 was assessed in male and female gametocyte functional viability assay, as reported by Ruecker et al. [22]. Briefly, gametocyte cultures were seeded at 1% rings and 4% haematocrit under 3% O_2_, 5% CO_2_, 92% N_2_ gas by using an asexual culture with 3% ring stages at day 0. Gametocyte cultures were tested for functional viability and maturity after 14 days. Testing functional viability was done by quantifying male gametocyte formation, which was carried out by withdrawing 200 μl of culture. Following this, culture was centrifuged, and the pellet was resuspended in 5 μl ookinete medium (RPMI medium with 25 mM HEPES, 50 mg/l hypoxanthine, 2 g/l sodium bicarbonate, 100 μM xanthurenic acid, and 10% A^+^ human serum). The culture was observed under a microscope. After validating maturity and upon exflagellation centres being > 50 per field, the gametocyte culture was resuspended in 7.5 ml complete medium. From this, 50 μl was dispersed into previously prepared 96-well plate (containing complete culture medium and FNDR-20123 at a concentration of 1 μM and prewarmed at 37 °C for 20 min). The plates were then incubated at 37 °C for 24 h. Gamete formation was induced on day 15 and observed under the microscope following the method described by Ruecker et al. [23]. The assay was performed using four independent biological replicates.

### In vitro killing profile

Killing profiles of FNDR-20123 was studied at GlaxoSmithKline’s Global Health Incubator Unit following a reported protocol [24]. Briefly, the assay used limiting dilution technique to quantify the number of parasites viable after drug treatment. *Plasmodium falciparum* strain 3D7 was treated with the selected compound FNDR-20123 and standard drugs (atovaquone, artemisinin, chloroquine, pyrimethamine) at a concentration corresponding to 10 × IC_50_. Parasites were treated for 10 h. The drugs used were renewed daily over the entire treatment period. Samples of parasites were taken from the treated culture every 24 h (0, for control of the initial number of parasites, 24, 48, 72, 96, 120 h’ time points). Following this, the procedure was carried out to wash out the drugs, and drug-free parasites were cultured in 96-well plates by adding fresh erythrocytes and new media (RPMI 1640 medium).

Three-fold serial dilution was used with the abovementioned samples after removing the drug to quantify the number of viable parasites after treatment. Parasites were cultured in microtitre plates to allow all wells with viable parasites to render detectable parasitaemia. Four independent serial dilutions were carried out with each sample to correct experimental variations. Samples were examined after 21 days of culturing. Additional sampling was done after 28 days to confirm the growth/no growth.

### Animals and treatment

Female NODscidIL2Rγ^null^ mice, weighing approximately 20–22 g, were used for the study. Mice were engrafted daily with 0.6 ml human blood intravenously (iv) in the tail (alternatively 0.8 ml intraperitoneally (ip) 11 days before the start of the study and were maintained in individually ventilated cages (IVC), but otherwise under standard conditions with 22 ℃ and 60–70% relative humidity, pellets (PAB45-NAFAG 9009, Provimi Kliba AG, CH-4303, Kaiseraugst, Switzerland) and water ad libitum. Mice were infected intravenously with parasitized red blood cells on day 0. The infected dose was 2 × 107 *P. falciparum*-infected erythrocytes at day 0. Experimental mice, n = 2 (NODscidIL2Rγ^null^ mice, females, 20–22 g) were treated at days 3, 4, 5, and 6 post-infection with an oral dose of the compound (4-day test by Peters) and were compared to an infected control group (n = 2 mice) for a reduction in parasitaemia on day 7.

### Single dose oral pharmacokinetics studies of FNDR-20123 in female SCID mice and male BALB/c mice

Female C.B-17/IcrHsd-Prkdc^scid^Lyst^bg^ mice (n = 3) aged 7-8 weeks and weighing around 20 to 22 g were used for experimentation after a minimum 7 days of acclimation. Male BALB/c mice (n = 3) aged 8–10 weeks and weighing around 20 to 22 g were also used for experimentation after a minimum 7 days of acclimation.

Fasted animals (3–4 h) were administered a single dose of test compound (FNDR-20123) by oral gavage with a dose of 50 mg/kg body weight in 0.1% (v/v) Tween 80 + 0.5% (w/v) methylcellulose at a dose volume of 10 mL/kg b.w. Feed was offered 2 h post-dose. Under mild isofluorane anaesthaesia, blood samples were collected by retro-orbital route into labelled centrifuge tubes containing K_2_EDTA anticoagulant at a concentration of 2 mg/mL of blood during the next 24 h post-dose. Collected blood samples were centrifuged at 4000 rpm for 10 min, and harvested plasma samples were stored at − 80 °C until analysis.

### In vivo efficacy

Therapeutic efficacy of FNDR-20123 in a SCID mouse model of human *P. falciparum* malaria was performed at Swiss Tropical and Public Health Institute (unit of Prof. Pascal Mäser). FNDR-20123 was dissolved in 100% DMSO and stored at − 20 °C until use. For the dose–response study, 50 mg of the compound was used in quintuplicate. Chloroquine (Sigma-Aldrich C6628) was used as the standard drug. The volume of 10 mL/kg was used for administration. Briefly, at day 0, in vitro cultures with approximately 2–5% parasitaemia were taken and diluted in culture medium to 2 × 10^7^ parasitized erythrocytes per 0.1 ml. This suspension was injected iv into experimental groups and a control group of n = 2 mice. From day 3 to 6 days post-infection, the experimental groups were treated with a single daily dose by the oral (po) route. Other routes, such as intramuscular and subcutaneous, were also tested using the same protocol. The drug concentration was adjusted in a way to administer 10 ml/kg. FNDR-20123 was also administered intraperitoneally at 50 and 100 mg/kg to humanized SCID mice infected with falciparum malaria. At 7th day post-infection, 2 μl tail blood was taken. The haematocrit was determined by FACS and parasitaemia by microscopy on > 10,000 red blood cells.

The difference of the mean infection rate of the control group (= 100%) to the test group was calculated and expressed as per cent reduction. As an example, activity determination with a mean of, e.g., 2% parasitaemia in treated mice and a mean of, e.g., 40% parasitaemia in the control animals was calculated as follows: (40–2%)/40% * 100 = 95% activity. The results were expressed as a reduction of parasitaemia at day 7 in  % as compared to the untreated control group. Values are expressed as mean ± SD.

### Repeated dose 14-day toxicity and toxicokinetics (TK) studies of FNDR-20123 in Sprague–Dawley rats

Toxicity and toxicokinetics (TK) studies of FNDR-20123 were performed at Antham Biosciences. Male and female Sprague–Dawley rats weighing roughly 200–250 g were assigned to control and different test groups of FNR-20123 (Table 1). The animal grouping was done by the method of body weight stratification and randomization. The animals procured for the study were weighed and grouped into body weight ranges. These body weight-stratified rats were distributed to all the study groups in equal numbers, such that body weight variation of animals does not exceed ± 20% of the mean body weight of each sex. Tween 80 (0.1% (v/v)) + methylcellulose (0.5% (w/v)) was used as a vehicle and the test item was dosed at a dose volume of 10 mL/kg bw. The animals were dosed once a day for a period of 14 consecutive days. The test item was administered at concentrations as mentioned in the Table for G2, G3, G4, G5 groups whereas vehicle was administered to G1 group (vehicle control) at the volume of 10 mL/kg body weight. Fasted animals from G5 were dosed on day 1 and day 14 and the blood samples were collected at specified time points (0.0, 0.08, 0.25, 0.5, 1.0, 2.0, 4.0, 8.0, and 24 h) post-dose. On days 2 to 13, animals were dosed in the fed state. During the study, observations were made for clinical signs and mortality, body weights, gross necropsy and organ weights. The data were subjected to statistical analyses using GraphPad Prism version 5.00, GraphPad Software. One way ANOVA with Dunnets post-test were done for different treatment groups compared with the control group data. All analyses and comparisons were evaluated at the 95% level of confidence (P < 0.05).

**Table 1:** Summary of animals subjected to 14-day toxicity study of FNDR-20123

Group no.	Dose of parent	Frequency of administration	Concentration (mg/mL)	Dose volume (mL/kg)	No. of animals sex/group
	(mg/kg bw)				
G1-Vehicle control	0.0	qd^a^	0.0	10	5
G2-Low dose	50.0	qd	5.0	10	5
G3-Mid dose	100.0	qd	10.0	10	5
G4-High dose	150.0	qd	15.0	10	5
G5-TK	100.0	qd	10.0	10	3

^a^qd–Once a day

## Results

### Screening the compounds as HDAC inhibitors

In the current study, a collection of proprietary compound library of HDAC inhibitors (n = 180) was screened for anti-malarial activity. A focus was placed on compounds which satisfied the criteria for inhibiting HDAC activity. FNDR HDAC inhibitors belong to four sub-chemotypes, and all four chemotypes show varying degrees of HDAC and anti-malarial activity (Table 2). These chemotypes selected are distinct from those published as the literature indicates that there are no HDAC inhibitors for malaria treatment pertaining to poor pharmacokinetics.

**Table 2:**
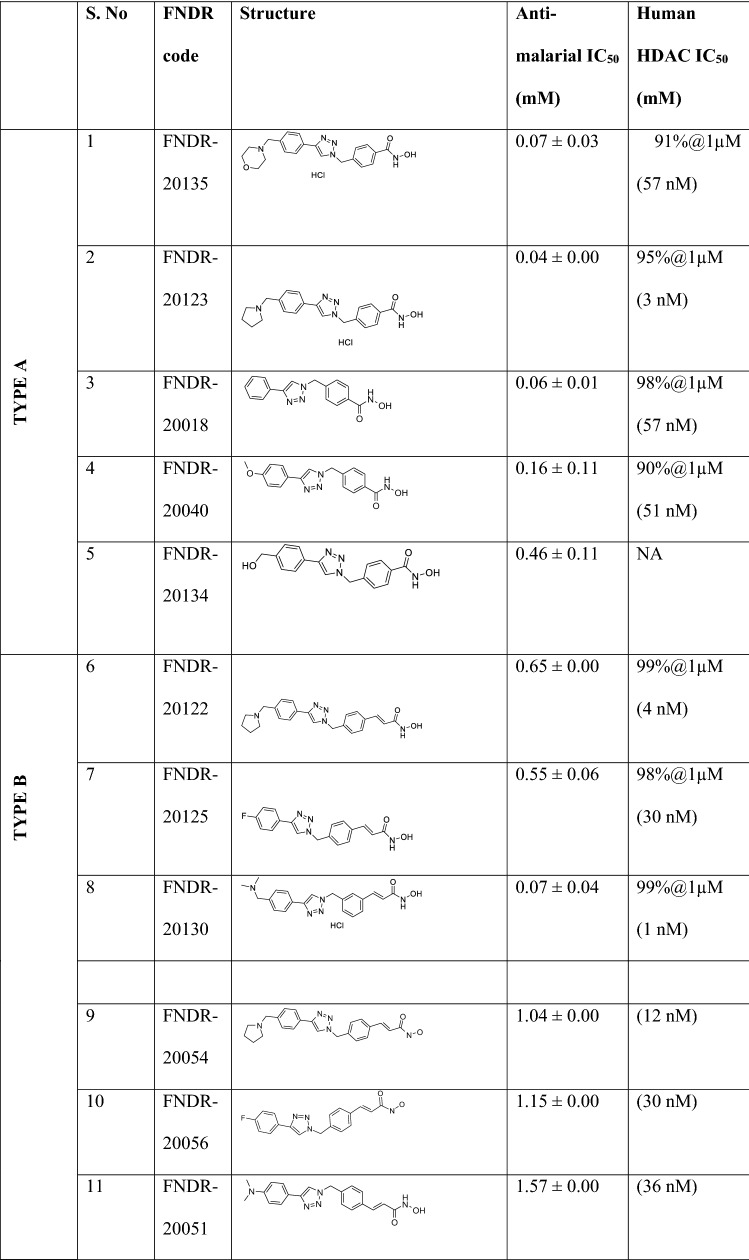

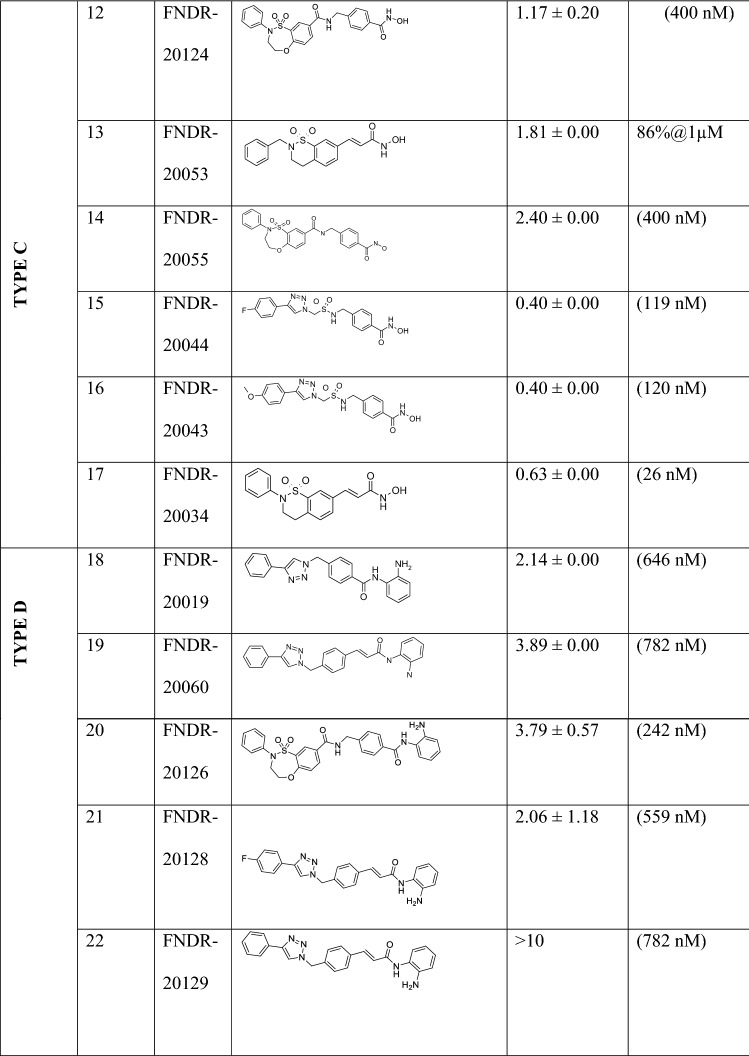
Summary of selected FNDR HDAC inhibitors belonging to four sub-chemotypes exhibiting anti-malarial activity with their structures

Isoform selectivity was also tested using recombinant HDAC isoforms in comparison with known HDAC inhibitor vorinostat (SAHA), serving as positive controls. Results indicated that FNDR-20123 is a potent inhibitor of HDAC at nanomolar concentrations. Isoform selectivity screening indicates the compound is a pan-HDAC inhibitor similar to vorinostat (Table 3).

**Table 3:** Isoform selectivity of FNDR-20123 tested against recombinant human HDAC isoforms

Compound	IC_50_ (nM)^a^					
	HeLa nuclear extract	HDAC1	HDAC2	HDAC3	HDAC6	HDAC8
Vorinostat	78	83	78	32	128	1612
FNDR-20123	3	25	29	2	11	282

^a^Single experiment assays

**Fig. 1:**
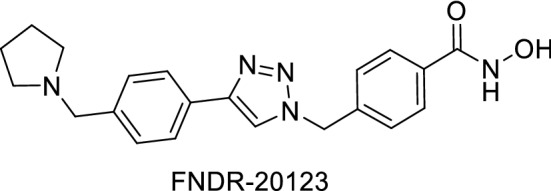
Molecular structure of FNDR-20123

The initial screening helped in identifying 10 active compounds (IC_50_ < 500 nM) which displayed *P. falciparum* inhibitory activity. For any drug to be made suitable for clinical use, it is imperative to select a compound which exhibits the highest potency; thus, the compounds were subjected to further analysis. From the selected candidates, FNDR-20123 (Fig. 1) exhibited potent anti-malarial activity inhibiting *Pf*HDAC1 and human HDAC with IC_50_s of 31 and 3 nM, respectively. Although the compound is a potent human HDAC inhibitor, a major challenge in the treatment of malaria is not anticipated since, the compounds are expected to be dosed for a short period (< 1 week) which does not affect normal human cells until a very high concentration is administered.

### Anti-malarial activity of FNDR-20123 against *Plasmodium falciparum* asexual stage and sexual blood stage

Anti-malarial activity of FNDR-20123 was determined by targeting the asexual blood-stage parasite *P. falciparum*. For this, a *P. falciparum* asexual blood stage assay was used, and the anti-malarial activity in asexual blood stage was found to be 41 nM. Following this, a gametocyte-functional viability assay was carried out for FNDR-20123 to investigate the anti-malarial activity of FNDR-20123 against the sexual stage of the parasite. The results obtained from the assay conducted in the study suggests that the compound FNDR-20123 exhibits gametocidal activity against male gametocytes with an IC_50_ of 190 nM (Table 4), thereby targeting the sexual blood stage of the parasite efficiently.

**Table 4:** Summary of physiological properties and anti-malarial activity of FNDR-20123

Compound	Physicochemical properties			Hu HDAC IC_50_ (nM)	*Pf*HDAC1	Anti-malarial activity	
	Mol. Wt.	ClogP	PSA	HeLa nuclear extract	IC_50_ (nM)	Asexual Blood Stage IC_50_ (nM)	Gametocytes IC_50_ (nM) Male/Female
FNDR-20123	377.45	1.917	80.53	3	31	41	190/> 5000

### In vitro killing profile

**Fig. 2:**
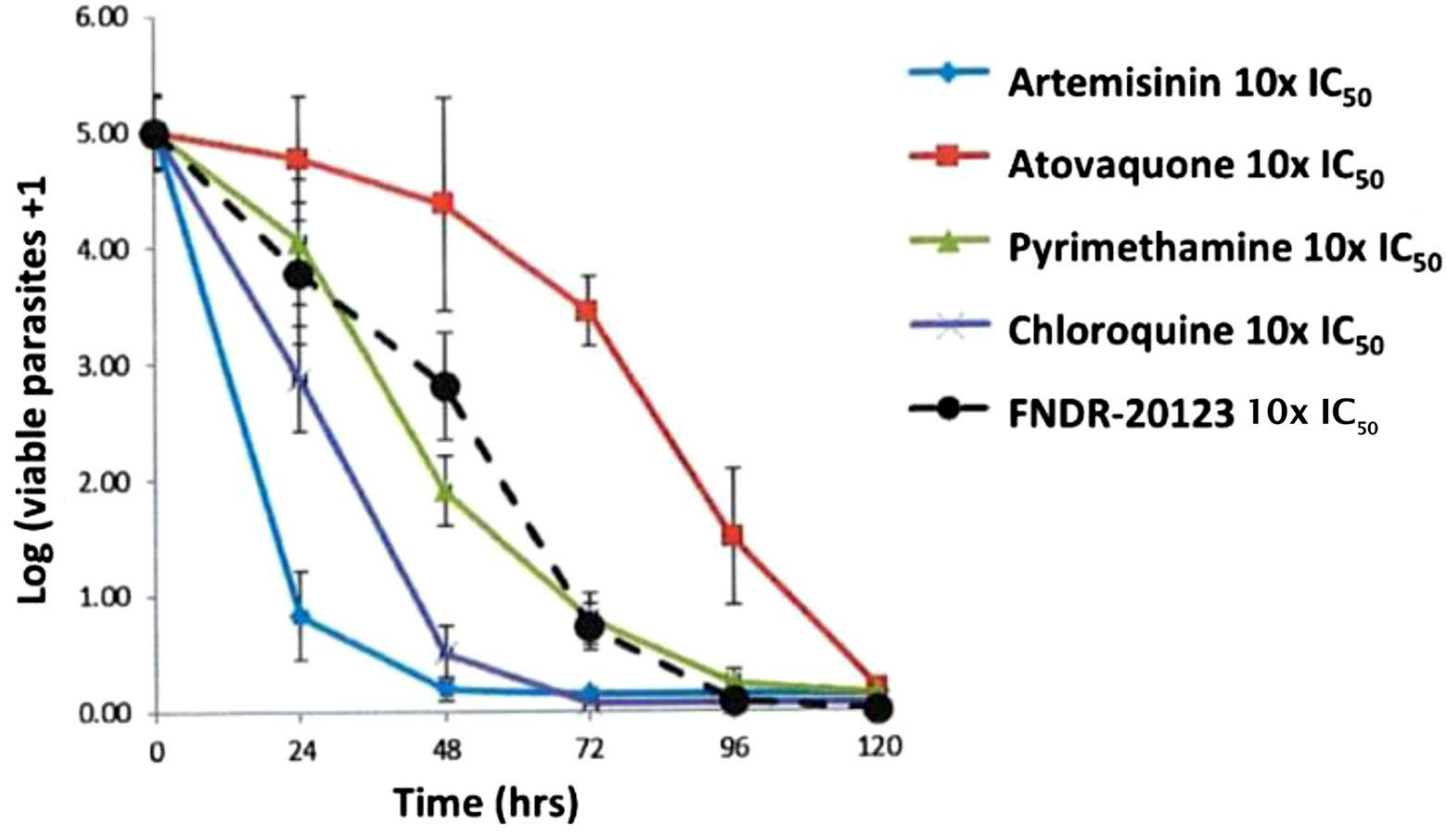
Killing profile of FNDR-20123 in comparison with artemisinin, atovaquone, pyrimethamine, and chloroquine at a concentration of 10 × IC_50_

The compound was further subjected to a comparative study wherein an in vitro killing profile of FNDR-20123 was studied in comparison to clinically available drugs: atovaquone, artemisinin, chloroquine, and pyrimethamine. When the selected compound was compared to the drugs, in vitro killing profiles of FNDR-20123 revealed that is better than atovaquone and comparable to pyrimethamine (Fig. 2), suggesting that the compound can be a faster killing drug than atovaquone.

### Safety profile of FNDR-20123

Upon testing the compound FNDR-20123 against all the mutants/strains, it was observed that the compound was sensitive in all the mutant/strains (Table 5a, b). From the results, it was observed that FNDR-20123 follows a distinct mechanism of action as it exhibited activity regardless of any resistance. In this study, different parasite strains/mutants were tested since each strain is known to display a separate resistance profile. Further, the safety of the compound FNDR-20123 was assessed in vitro. The results from the study depicted that the compound exhibited good liver microsomal stability, low plasma protein binding across species indicating increased metabolic stability (as the liver is the main organ of the body responsible for metabolism and detoxification), and increased availability and efficacy of the molecule. Drug-metabolising enzyme of the system, CYP450 and its isoform determines whether the compound will interact with other substances or not and are responsible for reactions such as reduction, hydrolysis and oxidation. Many drugs such as Propulsid, Lotronex, etc., have been discontinued from the market mainly due to interactions with other substances, determined by performing CYP inhibition assay [30]. Furthermore, when the compound was subjected to hERG (the human Ether-à-go-go-Related Gene) liability assay and CYP (Cytochrome) inhibition profiling, it was observed that the tested compound did not have any liability towards hERG channel and exhibited no inhibition of the CYP isoforms tested, thus rendering its profile safe (Table 6).

**Table 5:** (a) Cross-resistance profile of FNDR-20123 (b) Hypersensitivity profile of FNDR-20123

(a)	Mutated locus	Mutations (amino acid changes)		FNDR-20123	
				IC_50_ (nM)	Fold shift IC_50_ rel. Dd2
Dd2	Wild type	N/A		17.50	1.0
Dd2 DDD107498	*Pf*eEF2	Y186N		20.86	1.2
Dd2 390048	*Pf*pi4k	S743T		18.27	1.0
Dd2 DSM265	*Pf*dhodh	G181C		17.83	1.0
Dd2 GNF156	*Pf*carl	Ile1139Lys		18.45	1.1
Dd2 ELQ300	*Pf*cytB	Ile22Leu		13.49	0.8
(b)		FNDR-20123			
		IC_50_ (nM)	Fold shift IC_50_ rel. NF54		
NF54		25.27	1.0		
K1		19.05	0.75		
7G8		14.36	0.57		
TM90C2B		13.26	0.52		
Cam3.I (MRA1240)		22.06	0.87		
Dd2		17.50	0.69

**Table 6:** Safety profile of FNDR-20123

	FNDR-20123
Plasma protein binding (% bound)	
Mouse/Rat/Dog/Monkey/Human	< 30.00/41.89/< 20.00/< 20.00/57.30
Microsomal stability (% remaining at 120 min)	
MLM/RLM/HLM (Test Conc. 10 µM)	76.15/74.56/85.31
Plasma stability	
Mouse/Rat/Dog/Human (% remaining at 240 min)	89.16/88.90/97.72/98.94
CYP inhibition (IC_50_ µM)	
CYP 1A2	> 60
CYP 2C9	> 60
CYP 2C19	> 60
CYP 2D6	28.00
CYP 3A4	27.00
CYP induction (% induction relative to respective positive controls)	
CYP 1A2	2.8 ± 0.8
CYP 2B6	0.0
CYP 3A4	0.9 ± 1.5
Cytotoxicity	
HepG2 (% @ 100 µM)	12.6%
THP-1 (IC_50_ µM)	113.6 µM
hERG binding	
IC_50_ in µM	> 100 µM

Values are expressed as Mean ± SD

### Single dose oral pharmacokinetics studies of FNDR-20123 in female SCID mice and male BALB/c mice

**Fig. 3:**
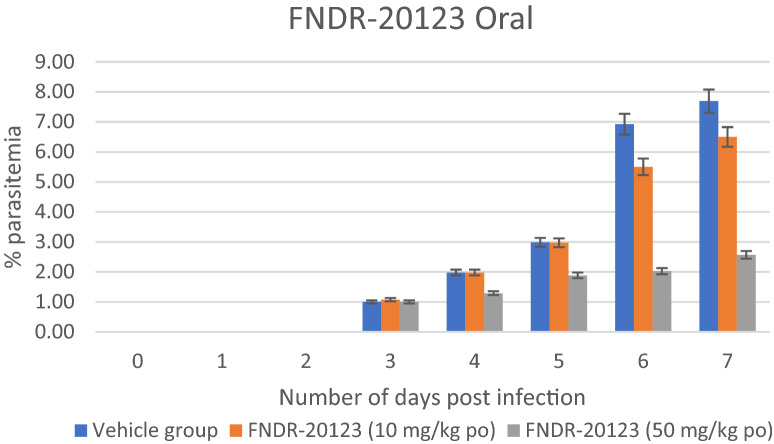
Therapeutic efficacy in the SCID mouse model of human falciparum malaria

The blood levels of the test compound are evaluated to determine standard pharmacokinetic parameters in the selected animal model of the efficacy study. The in vivo pharmacokinetic assays allow the quantitative evaluation of the time course of absorption, distribution, metabolism, and elimination (ADME) of a new substance. Pharmacokinetic analysis of the blood/plasma samples from the in vivo study revealed sub-optimal levels of the compound when dosed orally at these doses (Table 7). However, a significant increase in exposure was observed for FNDR-20123 when dosed subcutaneous and intramuscular at 50 mg/kg (Table 5). Levels over *Pf*HDAC IC_50_ have sustained up to ~ 15 h for both intramuscular (IM) and subcutaneous (SC) routes. Several fold increase in maximum drug concentration (Cmax (76x), area under the curve (AUC (33×) and half-life (t_1/2_ (2 to 9.21 h)) clearly indicates that FNDR-20123 will show enhance anti-malarial activity when dosed through subcutaneous route. The observed higher t_1/2_ also means once-a-day dosing regimen will be enough to achieve efficacy. Encouraged by the enhanced pharmacokinetic profiles observed by IM and SC routes, FNDR-20123 was administered intraperitoneally at 50 and 100 mg/kg to humanized SCID mice infected with *P. falciparum* malaria. Compared to the vehicle control (8.43%), FNDR-20123-treated group showed 0.19% (100 mg/kg bw) and 0.12% (50 mg/kg bw) parasitaemia after 4 days of administration (Fig. 3). There are no reports in the literature of the use of HDAC inhibitors in the SCID mouse model, to the best of the authors’ knowledge.

**Table 7:** Pharmacokinetic parameters of FNDR-20123

	Infected	Normal	Normal	Normal
Animal, strain	SCID mice	Balb/C mice	Balb/C mice	Balb/C mice
Route of administration	Oral	Oral	Subcutaneous	Intramuscular
Dose (mg/Kg)	50	50	50	50
T_max_ (h)	1.00	1.00	0.08	0.08
Cmax (ng/mL)	460	184	14039	15654
AUC_last_ (h*ng/mL)	1340	327	10,981	7224
t_1/2_ (h)	Not calculated	2.02	9.21	8.72

Values are expressed as Mean ± SD

N = 3 mice

### In vivo efficacy

In vivo efficacy study was conducted at Swiss Tropical and Public Health Institute in a humanized SCID mouse model of falciparum malaria. This study aimed to evaluate the therapeutic efficacy of FNDR-20123 against *P. falciparum Pf3D7*^0087/N9^ in NODscidIL2Rγ^null^ mice engrafted with human erythrocytes. The efficacy estimated in this study was expressed as the reduction (in  %) of parasitaemia. FNDR-20123 was dosed orally at 10 and 50 mg/kg bw for 4 days.

**Fig. 4:**
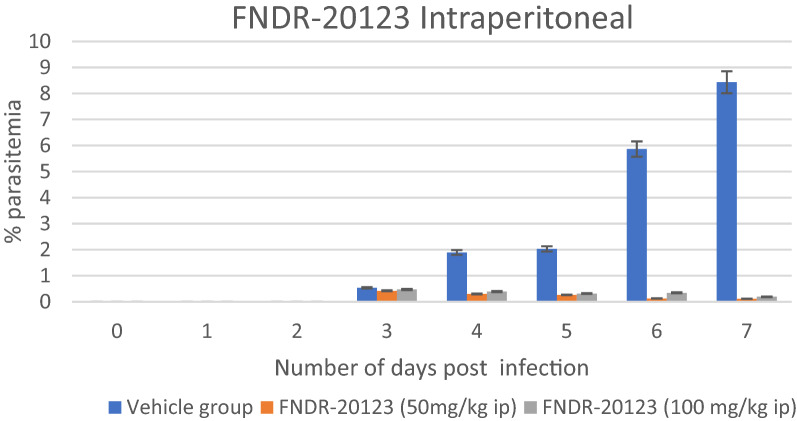
Therapeutic efficacy in SCID mouse model of human falciparum malaria (n = 2 mice per group)

While the vehicle-treated group showed 7.69% parasitaemia, oral administration of FNDR-20123 with 10 and 50 mg/kg bw resulted in a significant reduction in parasitaemia with 6.5% and 2.57% parasitaemia, respectively, from days 4 to 7 (Fig. 4). The study has used the humanized SCID mouse model, and n = 2 has been used for each group. The Swiss Tropical and Public Health Institute, Basel, where the study was conducted, has enough historical data to suggest that n = 2 is sufficient to enable a decision on the efficacy of the molecule in this mouse model.

### Repeated dose 14-day toxicity and toxicokinetics (TK) studies of FNDR-20123 in Sprague–Dawley rats

A repeat dose 14-day toxicokinetic study of FNDR-20123 by oral gavage in male and female Sprague–Dawley rats was performed to obtain data for choosing doses for 28-day repeat-dose studies. The test intends to collect information about the possible health risks using the repeated exposure to the selected compound. Generally, changes in the body weight of the animals are used as indicators of any adverse effect of the drug. However, most studies report that the increase or decrease in the weight is associated with normal physiological adaptation response leading to accumulation of fat or cutting fat molecules [31], though higher doses may induce stress and progress towards stimulating toxicity. The study did not reveal any adverse clinical signs or mortality in both the sexes. The 14-day repeated dose oral administration of FNDR-20123 at three dose levels of 50, 100 and 150 mg/kg body weight had no adverse toxic effects on body weights and organ weights in male and female Sprague–Dawley rats at termination (Tables 8, 9, 10). TK studies are carried out to determine the in vivo ADME of the compound by evaluating the potential of a substance to accumulate in a specific organ and/or tissue. Systemic exposure of the compound assists in establishing the relationship between dose administration and time course of the compound in the system and pharmacokinetic parameter determination of a compound following administration of multiple doses enables the most accurate interpretation of the toxicological findings. Data obtained from this study, along with the toxicology studies, helps in determining the safety of the compound in humans when subjected to clinical studies. The results (toxicokinetic profile at dose 100 mg/kg bw) reveal no accumulation of FNDR-20123, suggesting that the compound is being cleared from the system within 24 h of administration by oral gavage in Sprague–Dawley rats (Table 11).

**Table 8:** Summary of body weights (g) in females and males (FNDR-20123)

Groups	Dose (mg/kg bw)	Female			Male		
		Day					
		1	7	14	1	7	14
G1	0	143.05	152.03	160.27	171.51	193.40	211.52
		11.43	23.65	22.62	14.25	15.86	21.33
G2	50	146.45	150.70	161.49	166.04	190.40	209.30
		6.69	4.32	4.76	11.77	7.67	10.54
G3	100	146.70	145.63	158.43	166.39	173.72	177.16
		13.79	15.68	14.58	16.21	26.92	41.74
G4	150	144.18	139.82	142.91	170.86	180.10	188.27
		10.00	14.44	20.97	10.31	11.32	12.22

G5-toxicokinetic group

Values are expressed as Mean ± SD

**Table 9:** Summary of Relative Organ Weights (g)–Males

Groups, sex and dose (mg/kg)	Fasting body weight (g) Mean ± SD	Brain	Heart	Liver	Kidney	Thymus	Spleen	Lungs	Testis	Epididymis
G1 M 0	191.68	0.92	0.35	4.02	0.91	0.13	0.48	0.96	1.45	0.30
	19.15	0.10	0.03	0.34	0.13	0.06	0.10	0.67	0.13	0.05
G2 M 50	189.75	0.91	0.36	4.13	0.88	0.09	0.38	0.69	1.44	0.26
	8.99	0.06	0.00	0.56	0.10	0.04	0.14	0.07	0.10	0.05
G3 M 100	163.82	1.25	0.46	5.62	1.12	0.10	0.80	0.99	1.94	0.28
	38.21	0.15	0.14	2.73	0.44	0.04	0.87	0.37	0.59	0.11
G4 M 150	174.95	0.98	0.35	3.72	0.91	0.10	0.21	0.76	1.54	0.34
	11.67	0.11	0.04	0.39	0.08	0.02	0.07	0.15	0.16	0.07

G5-toxicokinetic group

Values are in Mean ± SD; G1 vs G2, G3 & G4 (*p < 0.05)

**Table 10:** Summary of relative organ weights (g) (FNDR-20123)–females

Groups and dose (mg/kg)	Fasting body weight (g)	Brain	Heart	Liver	Kidney	Thymus	Spleen	Lungs	Uterus
G1 F 0	145.81	1.17	0.41	4.89	1.07	0.27	0.69	0.99	0.23
	21.82	0.12	0.05	0.17	0.19	0.04	0.27	0.23	0.09
G2 F 50	149.51	1.25	0.44	5.03	1.04	0.18	0.47	0.95	0.24
	5.23	0.26	0.13	1.27	0.23	0.06	0.19	0.34	0.08
G3 F 100	143.57	1.22	0.41	4.55	1.01	0.12*	0.38	0.80	0.15
	15.63	0.17	0.04	0.43	0.12	0.06	0.29	0.13	0.04
G4 F 150	134.25	1.28	0.42	4.74	1.03	0.16*	0.38	0.96	0.21
	21.07	0.23	0.02	0.45	0.20	0.05	0.16	0.43	0.09

G5-toxicokinetic group

Values are in Mean ± SD; G1 vs G2, G3 & G4 (*p < 0.05)

**Table 11:** Repeated dose 14-day toxicokinetics study in male and female Sprague–Dawley Rats

TK Parameters	FNDR-20123 (100 mg/kg bw)			
	Male		Female	
	Day 1	Day 14	Day 1	Day 14
C_max_ (ng/mL)	420.04 ± 174.42	702.66 ± 362.42	228.72 ± 69.32	206.96 ± 36.13
T_max_ (h)	0.33 ± 0.14	0.19 ± 0.10	0.28 ± 0.21	0.14 ± 0.10
AUC_last_ (h*ng/mL)	711.78 ± 233.81	956.67 ± 180.88	344.65 ± 71.63	480.15 ± 49.73
AUC_inf_ (h*ng/mL)	1278.86 ± 282.19	1075.02 ± 215.59	448.27 ± 112.83	573.42 ± 23.07
T_1/2_ (h)	5.72 ± 4.10	2.30 ± 0.55	3.21 ± 0.41	2.40 ± 0.26

## Discussion

In India, malaria is considered a public health problem. Most malaria cases are reported from the eastern and central part of the country and from states which have forest, hilly and tribal areas. In India, malaria cases have consistently declined from 2.08 million in 2001 to about 0.4 million in 2018 [1]. However, the emergence of anti-malarial drug resistance necessitates the development of novel anti-malarials. In accordance with this, a new class of chemical compounds known as HDAC inhibitors were was identified with a potential to target *Plasmodium* and other Apicomplexan parasites more than a decade ago.

The sexual stage of the parasite is an obligatory link continuing the life cycle of the parasite in the vector. To date, asexual blood stage of the malarial parasite has been considered a druggable target for most anti-malarial drugs. However, severing the transmission link by targeting the sexual stage appears to be critical in progressing towards malaria elimination. It is considered to be the new ‘druggable target’ of interest as gametocytes in the sexual stage of the parasite develop through 5 morphologically discrete stages in the human blood over 8 to 12 days, which thereafter persist in the peripheral blood for weeks [25]. Additionally, while most scientists have often focused on resolving disease symptoms by targeting the asexual blood stage of the parasite [26], there has been a considerable increase in developing drugs with the ability to target the sexual stage of the parasite, i.e., the gametocyte stage, as this is the most critical and accessible developmental stage for transmission-blocking drugs [27]. Targeting the gametocyte stage of the parasite limits the chances of transmission of resistant parasite strains. The study indicates that the compound FNDR-20123 is a potent inhibitor of both asexual and sexual stages of the parasite.

Upon further subjecting the compound to in vitro killing profile, FNDR-20123 was studied in comparison to clinically available drugs. Amongst the available drugs, artemisinin (fast-killing drugs) is known to have the strongest killing profile against *P. falciparum*, achieving up to 99.99% decrease in parasitaemia during one life cycle [24]. Most reports suggest that differences in the killing profiles exist due to the respective chemosensitivity of developing gametocytes and asexual stages to anti-malarial compounds such as chloroquine, pyrimethamine, atovaquone [28]. The study indicates that the compound FNDR-20123 is a faster killing drug than atovaquone, which is known to be a cytostatic drug that might fail to eliminate circulating parasites leading to treatment failure and resistance selection [24].

The safety of the compound FNDR-20123 is assessed in vitro before it is progressed for pre-clinical trials to predict for any side effects which may occur and to determine the ADME fate of the molecule in vivo by analysing the factors that determine the pharmacokinetic profile of the compound. Safety of a compound is determined by measuring its physiological parameters, such as plasma protein binding, liver microsomal stability, hERG liability, and CYP inhibition [29]. These parameters help in addressing questions such as what per cent of the compound plasma protein is bound, to which component (sub-fraction), and what will be the free fraction available to cover the target, how long will the lead molecule circulate in plasma within the body, whether the compound inhibits a key oxidative metabolic enzyme that will lead to subsequent drug–drug interactions [30]. The compound FNDR-20123 was tested for the parameters and exhibited properties which rendered it safe.

Once the safety of a compound is assessed and deemed fit, the compound is further progressed for a repeat dose 14-day toxicokinetic study, which is mainly intended to collect information about the possible health risks using the repeated exposure to the selected compound. Generally, changes in the body weight of the animals are used as indicators of any adverse effect of the drug. However, most studies report that the increase or decrease in the weight is associated with a normal physiological adaptation response leading to accumulation of fat or cutting fat molecules [31], although higher doses may induce stress and progress towards stimulating toxicity. The compound FNDR-20123 did not reveal any adverse clinical signs or mortality, which suggests that no possible health risk will be associated.

The toxicology and toxicokinetic studies indicate that the compound is safe when administered via the oral route. Investigational new drug (IND) enabling toxicology studies with the systemic route of administration are planned with FNDR-20123.

Data obtained from this study, along with the toxicology studies, suggest that the compound will be safe in humans when subjected to clinical studies.

## Conclusion

Globally, malaria puts 2 billion people at risk claiming approximately 5 lakh deaths each year. In view of this, the proposed treatment method involved using HDAC inhibitors to target the regulatory enzymes (HDAC) responsible for post-translational modifications (causing various human diseases). The results identified FNDR-20123 as a potent, safe and a first-in-class anti-malarial HDAC inhibitor active against all resistant strains tested so far, which will be highly valuable in eliminating the rapidly evolving drug-resistant parasite. The development of the compound FNDR-20123 into a drug will be an improvement from the other compounds which could not be developed for clinical trials. FNDR 20123 has relatively better potency and bioavailability and may provide a more cost-effective option, benefitting a large population of malaria-infected patients. The authors’ believe that an HDAC inhibitor will mechanistically combine well with other known anti-malarial drugs and will act against drug-resistant strains, benefitting patients affected with resistant *P. falciparum* which has now been emerging in various parts of India.

## Data Availability

The datasets generated during the current study are available from the corresponding author on reasonable request.
